# Supplementary material for: Comprehensive Annotation and Functional Exploration of MicroRNAs in Lettuce
Source: Front Plant Sci. 2021 Dec 24;12:781836. doi: 10.3389/fpls.2021.781836 (PMC8739914; doi:10.3389/fpls.2021.781836)
Supplement: Supplementary file 3 [file Data_Sheet_9.ZIP › GSM2230753.plot/Lsa-miR396e_Lsat_1_v5_gn_8_127960.1_2426_TPlot.pdf]

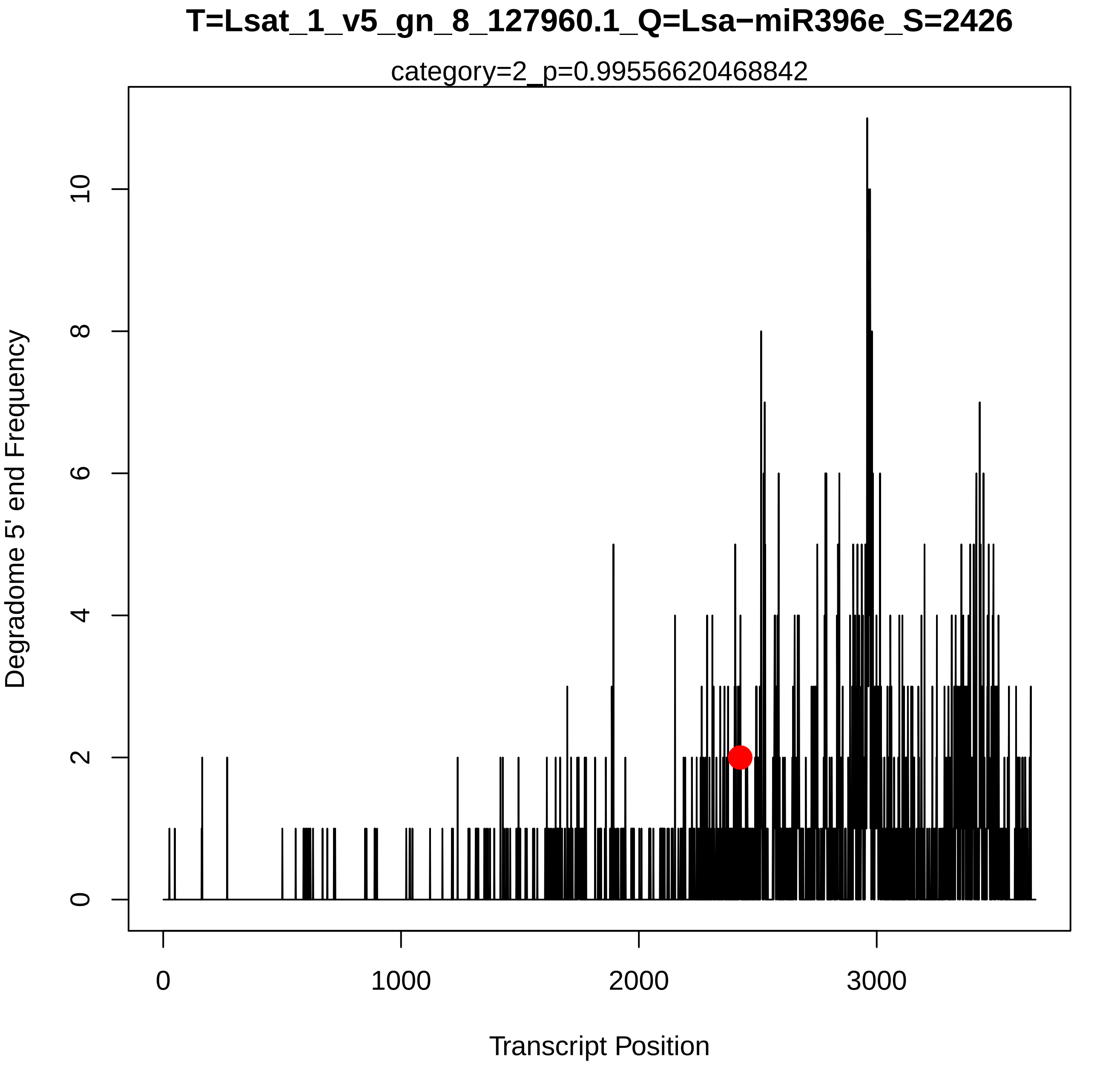

T=Lsat_1_v5_gn_8_127960.1_Q=Lsa−miR396e_S=2426
category=2_p=0.99556620468842
Degradome 5' end Frequency
0
2
4
6
8
10
0
1000
2000
3000
Transcript Position
